# The mechanism by which moderate alcohol consumption influences coronary heart disease

**DOI:** 10.1186/s12937-015-0011-6

**Published:** 2015-04-02

**Authors:** Marc J Mathews, Leon Liebenberg, Edward H Mathews

**Affiliations:** CRCED, North-West University, and Consultants to TEMM International (Pty) Ltd, P.O. Box 11207, Silver Lakes, 0054 South Africa

**Keywords:** Coronary heart disease, Moderate alcohol consumption, Biomarkers

## Abstract

**Background:**

Moderate alcohol consumption is associated with a lower risk for coronary heart disease (CHD). A suitably integrated view of the CHD pathogenesis pathway will help to elucidate how moderate alcohol consumption could reduce CHD risk.

**Methods:**

A comprehensive literature review was conducted focusing on the pathogenesis of CHD. Biomarker data were further systematically analysed from 294 cohort studies, comprising 1 161 560 subjects. From the above a suitably integrated CHD pathogenetic system for the purpose of this study was developed.

**Results:**

The resulting integrated system now provides insight into the integrated higher-order interactions underlying CHD and moderate alcohol consumption. A novel ‘connection graph’ further simplifies these interactions by illustrating the relationship between moderate alcohol consumption and the relative risks (RR) attributed to various measureable CHD serological biomarkers. Thus, the possible reasons for the reduced RR for CHD with moderate alcohol consumption become clear at a glance.

**Conclusions:**

An integrated high-level model of CHD, its pathogenesis, biomarkers, and moderate alcohol consumption provides a summary of the evidence that a causal relationship between CHD risk and moderate alcohol consumption may exist. It also shows the importance of each CHD pathway that moderate alcohol consumption influences.

## Background

The World Health Organisation indicates coronary heart disease (CHD) as the leading cause of death globally [1]. It is also well documented that moderate alcohol (ethanol) consumption is associated with a lower relative risk of CHD events [2-9]. However, the precise integrated mechanisms of this lower risk are not always clear at a glance.

Possible mechanisms may be due to the direct actions of alcohol on specific pathogenetic pathways of CHD which can be measured via serological biomarkers of CHD. Typically elevations of high density lipoprotein (HDL) cholesterol levels, increases in serum adiponectin levels [10], reduction in C-reactive protein (CRP) serum levels [11], reduced serum fibrinogen levels [12], and increased insulin sensitivity [13] have all been suggested as possible positive influences of moderate alcohol consumption. However, we are not sure of the relative importance of each suggestion and if these are the only influences.

Therefore, the purpose of this study is to visually integrate information on how moderate consumption of alcohol influences the pathogenesis of CHD. These influences may then provide a useful integrated summary for the plausibility of a causal relationship between moderate alcohol consumption and a reduction in CHD risk.

## Methods

The integrative view of CHD is relevant to many other health issues such as diet, depression, stress, insomnia, sleep apnoea, exercise, smoking and oral health. For full comprehension of the effect of alcohol it is replicated here from [14].

### Search criteria

We searched PubMed, Science Direct, Ebsco Host, and Google Scholar for publications with “coronary heart disease“or “coronary artery disease” or “cardiovascular disease” or “CHD” as a keyword and combinations with “lifestyle effects”, “relative risk prediction”, “network analysis”, “pathway analysis”, “interconnections”, “systems biology”, “pathogenesis”, “biomarkers”, “conventional biomarkers”, “hypercoagulability”, “hypercholesterolaemia”, “hyperglycaemia”, “hyperinsulinaemia”, “inflammation”, and “hypertension” in the title of the study.

We also searched all major relevant specialty journals in the areas of cardiology, nutrition, alcohol consumption, endocrinology, psychoneuroendocrinology, systems biology, physiology, CHD, the metabolic syndrome and diabetes, such as *Circulation; Journal of the American College of Cardiology; Arteriosclerosis, Thrombosis and Vascular Biology; The Lancet; New England Journal of Medicine; American Journal of Medicine; Nature Medicine; Diabetes Care; Journal of Clinical Endocrinology and Metabolism; American Journal of Clinical Nutrition;* and *Journal of Physiology* for similar or related articles.

Furthermore, we selected PubMed and Google Scholar for meta-analyses with keywords “coronary heart disease” or “coronary artery disease” or “cardiovascular disease” or “CHD”. We also reviewed articles referenced in primary sources and their relevant citations. However, unless cited more than 50 times, we included only articles published after 1998 as these contained the most significant data.

### Study selection

Only the trends from each meta-analysis that was adjusted for the most confounding variables was used and only where sufficient information was available on that trend. This was done so that the effects of most of the potential confounders could be adjusted for. This may, however, have increased the heterogeneity between studies, as not all studies adjusted for the same confounders.

CHD was classified as the incidence of atherosclerosis, coronary artery disease, or myocardial infarction. Where results were given for cardiovascular disease these were interpreted as CHD only in scenarios where the effect of stroke could be accounted for or results were presented separately. Biomarkers were only considered if they were associated with an increased or decreased risk of CHD.

The RR data for changes in biomarker levels were extracted from the relevant publications. However, it was not the intention of this study to conduct individual meta-analyses of the individual biomarkers. Thus the RR for changes in biomarkers were, where possible, extracted from the most recent meta-analysis conducted on the specific biomarker. If no meta-analysis was available, a suitable high quality study was included. In order to limit errors in comparisons between separate biomarkers only RR values given per increase of 1-standard deviation (SD) in the biomarker level were included. This standardisation of RR to RR per 1-SD prohibits the misrepresentation of risk due to the selection of extreme exposure contrasts [15].

### Data analysis

Heterogeneity between studies was inevitable due to the large quantity of meta-analyses considered. Each underlying meta-analysis reported individually on the heterogeneity in their analysis. However, these effects were not so large as to discount the effects observed.

The individual meta-analyses also had detailed accounts of differences between studies and subgroup analyses. These aspects are not further elaborated on in this study as they were used as a measure of validity in the study inclusion process. The individual studies selected unfortunately represent only the risk associated with the cohort studied and cannot be accurately extrapolated to other populations without further research.

OR and HR were converted to RR using the approach outlined by Zou [16]. It must however be noted that some of the RR values in this article differ from convention. The need for this comes as a result of the visual scaling of the traditional RR. Traditionally, if one plots an RR = 3 and RR = 0.33, respectively, the one does not ‘look’ three times worse and the other three times better than the normal RR = 1. The reason is that the scales for the positive and negative effects are not numerically similar. A graph of ‘good’ and ‘bad’ RR can therefore be deceptive for the untrained person, e.g., a patient.

This article rather uses the method that the conventional RR = 3 is three times worse than the normal RR = 1. While the conventional RR = 0.33 means that the patient’s position is three times better than the normal RR = 1. Thus, in summary: a conventional RR = 3 is presented as per normal, as a 3-fold increase in risk and a conventional RR = 0.33 is presented as a 3-fold decrease in risk (1/0.33 = 3).

## Results and discussion

### Integrated view of coronary heart disease

An investigation of the interconnectivity of lifestyle factors (and specifically of moderate alcohol consumption), CHD pathogenesis, and pathophysiological traits attributed with the disorder was conducted. This study was based on data extracted from published metastudies, where genetic risk factors for CHD were not considered.

**Figure 1:**
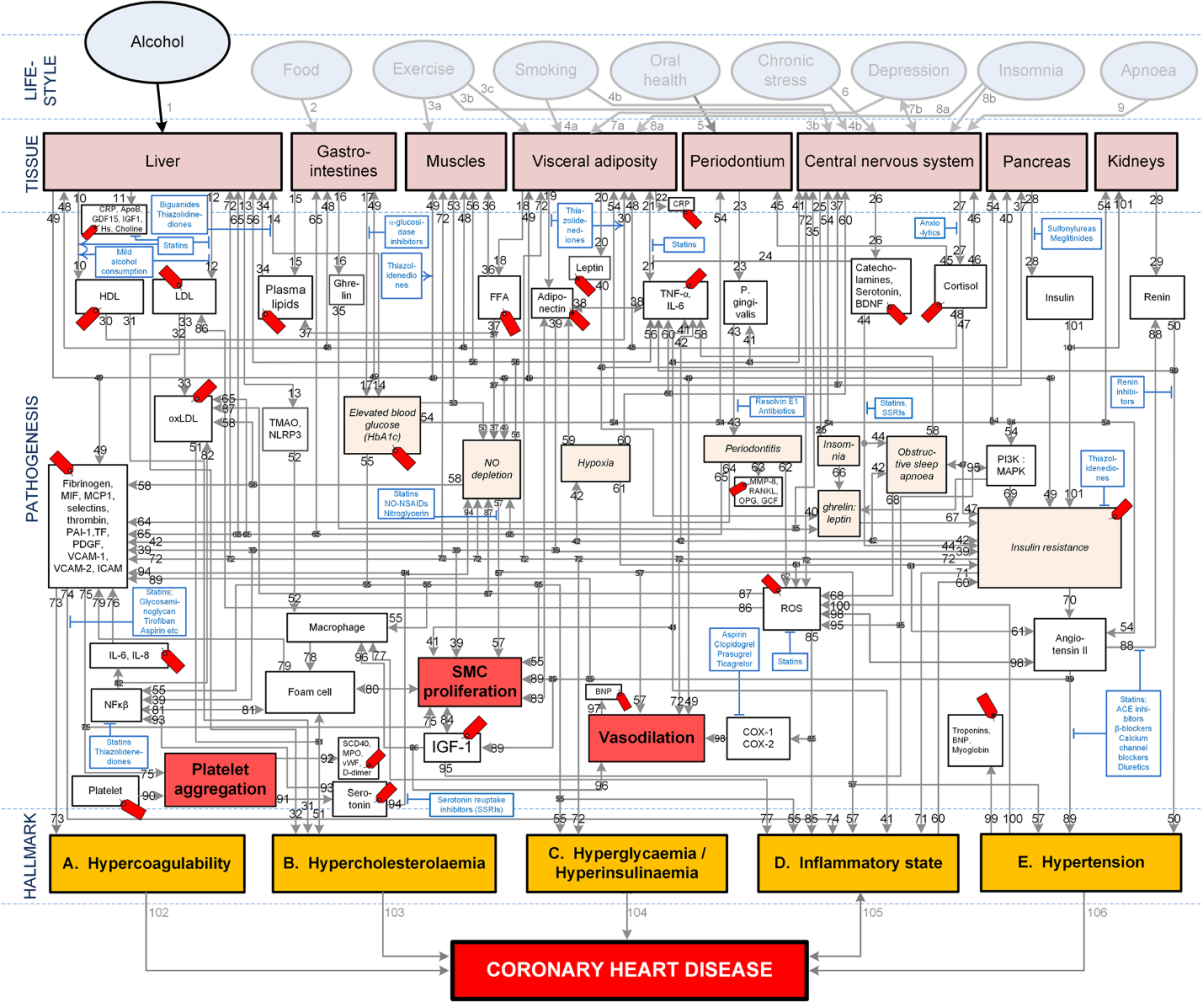
**Conceptual model of general lifestyle factors, salient CHD pathogenetic pathways and CHD hallmarks.** From “How do high glycemic load diets influence coronary heart disease?” by Mathews MJ, Liebenberg L, Mathews EH. Nutr Metab. 2015:12:6 [14]. The affective pathway of pharmacotherapeutics, blue boxes, is shown in Figure 1, and salient serological biomarkers are indicated by the  icon. The blunted blue arrows denote antagonise or inhibit and pointed blue arrows denote up-regulate or facilitate. HDL denotes high-density lipoprotein; LDL, low-density lipoprotein; oxLDL, oxidised LDL; FFA, free fatty acids; TMAO, an oxidation product of trimethylamine (TMA); NLRP3, Inflammasome responsible for activation of inflammatory processes as well as epithelial cell regeneration and microflora; Hs, homocysteine; IGF-1, insulin-like growth factor-1; TNF-α, tumour necrosis factor-α; IL, interleukin; NO, nitric oxide; NO-NSAIDs, combinational NO-non-steroidal anti-inflammatory drug; SSRI, serotonin reuptake inhibitors; ROS, reactive oxygen species; NFκβ, nuclear factor-κβ; SMC, smooth muscle cell; HbA_1c_, glycated haemoglobin A_1c_; P. gingivalis, Porphyromonas gingivalis; vWF, von Willebrand factor; PDGF, platelet-derived growth factor; MIF, macrophage migration inhibitory factor; SCD-40, recombinant human sCD40 ligand; MPO, myeloperoxidase; MMP, matrix metalloproteinase; VCAM, vascular cell adhesion molecule; ICAM, intracellular adhesion molecule; CRP, C-reactive protein; PAI, plasminogen activator inhibitor; TF, tissue factor, MCP, monocyte chemoattractant protein; BDNF, brain-derived neurotrophic factor; PI3K, phosphatidylinositol 3-kinase; MAPK, mitogen-activated protein (MAP) kinase; RANKL, receptor activator of nuclear factor kappa-beta ligand; OPG, osteoprotegerin; GCF, gingival crevicular fluid; D-dimer, fibrin degradation product D; BNP, B-type natriuretic peptide; ACE, angiotensin-converting-enzyme; COX, cyclooxygenase; β-blocker, beta-adrenergic antagonists.

A suitably integrated CHD model of the pathogenesis and serological biomarkers of moderate alcohol consumption was not found in the literature. Such a model was thus developed and is presented in Figure 1, which schematically illustrates the complexity of CHD [14].

It is however important to realize that CHD involves inputs from hundreds of gene expressions and a number of tissues. Thus, when investigating CHD, analysing the individual components of the system would not be sufficient, as it is also important to know how these components interact with each other [17]. For instance, genetic and lifestyle factors influence clinical traits by perturbing molecular networks [18]. A high-level systems-based view of CHD therefore has the potential to interrogate these molecular phenotypes and identify the patterns associated with the disease.

Pathways can be tracked from a chosen lifestyle factor to a hallmark of CHD if the two states are connected by the pathogenesis of the disorder. The pathways are therefore a visual representation of previously published knowledge merely integrated here. The pathogenetic pathways of interest for this review were only those between moderate alcohol consumption and CHD.

The lifestyle factor of “Alcohol” (Figure 1) was regarded as comprising of a moderate intake of 20–30 g of alcohol (ethanol) per day for men and half that for women [9]. “Tissue” in Figure 1 indicates the organ or type of tissue which is affected by a pathogenetic pathway or trait. “Pathogenesis” in Figure 1 indicates the pathogenetic pathways of the disorder.

Salient serological biomarkers (shown in Figure 1 as ) and pharmacotherapeutics (shown in Figure 1 as ) that act on the pathways are also indicated in Figure 1. These pathogenetic pathways also lead to certain traits (e.g. insulin resistance) that lead to five pathophysiological end-states, which we designate as “hallmarks of CHD”, namely hypercoagulability, hypercholesterolaemia, hyperglycaemia/hyperinsulinaemia, an inflammatory state, and hypertension.

The formulation of this conceptual model required the consultation of numerous publications. The journal references which were used to describe the main pathogenetic pathways in the model are given in Table 1 [14]. It is however not the purpose of this review to describe in detail all these pathways. The aim is merely to simplify Figure 1 to elucidate only the pathways relevant to moderate alcohol consumption.

**Table 1:** **Pathogenetic pathways (in Figure** **1** **) and cited works**

Pathway	Refs.	Pathway	Refs.	Pathway	Refs.	Pathway	Refs.	Pathway	Refs.	Pathway	Refs.
**1**	[4,44]	**2**	[45-49]	**3 a,b,c**	[50-52]	**4 a,b**	[53-55]	**5**	[56-58]	**6**	[59-61]
**7 a,b**	[62-67]	**8 a,b**	[68-70]	**9**	[71]	**10**	[30,72-75]	**11**	[19,30]	**12**	[30]
**13**	[47-49]	**14**	[19,76-83]	**15**	[82-84]	**16**	[68-70]	**17**	[76-83]	**18**	[55,85-87]
**19**	[84,85]	**20**	[68-70]	**21**	[19,27,28,87-91]	**22**	[87]	**23**	[92-96]	**24**	[97-99]
**25**	[68-70]	**26**	[97-102]	**27**	[34,60,61,103-114]	**28**	[115-119]	**29**	[30,120]	**30**	[19,30,72-75]
**31**	[19,30,72-75]	**32**	[30]	**33**	[30]	**34**	[19,84-87]	**35**	[68-70]	**36**	[19,84-87]
**37**	[19,84-87]	**38**	[28,34,121-125]	**39**	[84,85]	**40**	[68-70]	**41**	[27,28,125]	**42**	[27,119]
**43**	[27,28,56,92-96]	**44**	[97-99]	**45**	[59,107,109]	**46**	[59,107,109]	**47**	[59,107,109]	**48**	[59,107,109]
**49**	[115-117,126]	**50**	[30,122,127]	**51**	[17-19,21,30,120,121,127-130]	**52**	[47,48]	**53**	[19,76-83]	**54**	[19,76-83]
**55**	[19,76-83,131-136]	**56**	[19,84-87]	**57**	[19,84-87,120,137-140]	**58**	[19,84-87,120]	**59**	[110-113]	**60**	[110-113]
**61**	[110-113]	**62**	[56,93]	**63**	[92-95]	**64**	[56,57]	**65**	[56,57,94]	**66**	[68-70]
**67**	[68-70]	**68**	[84-87]	**69**	[115]	**70**	[115-117]	**71**	[30,115-117,120,141,142]	**72**	[30,115-117,120,141,142]
**73**	[19,72,119]	**74**	[19,72,119]	**75**	[72,91,119,131,138]	**76**	[19,27,28]	**77**	[27,131]	**78**	[27,131]
**79**	[19,27,72,131]	**80**	[19,27,72,131]	**81**	[27,72,131]	**82**	[72,115,121]	**83**	[133-136]	**84**	[27]
**85**	[19,121,128,139,140]	**86**	[19,121]	**87**	[121]	**88**	[30,121,138,141,142]	**89**	[30,121,138,141,142]	**90**	[72,131,138]
**91**	[97-99]	**92**	[30,72,129]	**93**	[97,98]	**94**	[143-146]	**95**	[147-150]	**96**	[147-150]
**97**	[30]	**98**	[27,72,121,131]	**99**	[30]	**100**	[121]	**101**	[115-117]	**102**	[30,76,79,115,121,129]
**103**	[19,27,30,90]	**104**	[19,30,121,129,138]	**105**	[19,27,30,90,151,152]	**106**	[19,30,121,129,138]				

From “How do high glycemic load diets influence coronary heart disease?” by Mathews MJ, Liebenberg L, Mathews EH. Nutr Metab. 2015:12:6 [14]. a, b, c denote the multiple pathways between lifestyle effects and CHD pathogenesis.

Despite the rich body of existing knowledge pertaining to CHD pathogenesis, lifestyle factors, and pharmacotherapeutics [17-21], a suitable integrated high-level conceptual model of CHD could not be found for the purpose of this study. A high-level model that consolidates the effects of moderate alcohol consumption on the RR of CHD and CHD biomarkers was thus developed. This model could thus help elucidate the higher-order interactions underlying CHD [17] and provide new insights into the relationship between CHD incidence and moderate alcohol consumption.

### Pathogenetic effects of moderate alcohol consumption

Figure 1 indicates all possible pathogenetic pathways between the considered lifestyle factors and CHD. In the current review only the CHD effects of moderate alcohol consumption, detailed in Table 2, are appraised. The pathogenetic pathways which are activated by moderate alcohol consumption are elucidated therein. It is important to note that not all the pathogenetic pathways indicated in Figure 1 will be relevant in all patients, and all the pathways may not be active simultaneously.

**Table 2:** **Putative effects of high-glycemic load diets and salient CHD pathogenetic pathways**

*Lifestyle*	*Pathways, and pathway numbers corresponding to those in Figure* *1*	*Refs.*
Moderate alcohol consumption	a. 1-12-↓ LDL-33-51-↓ hypercholesterolaemia	a. [3,7,8,25,26,153]
	b. 1-10-↑ HDL-31-↓ hypercholesterolaemia	b. [3,7,8,25,26,153]
	c. 1-14-↓ blood glucose-55-↓ hyperglycaemia	c. [3,7,8,25,26,153]
	d. 1-14-↓ blood glucose-54-69-↓ insulin resistance-70-89-↓ hypertension-100-↓ ROS-85-↓ inflammatory state	d. [28,153]
	e. 1-14-↓ blood glucose-54-69-↓ insulin resistance-72-↑ vasodilation	e. [128]

↑ denotes up regulation/increase, ↓ denotes down regulation/decrease, *x-y-z* indicates pathway connecting *x* to *y* to *z*. HDL, high-density lipoprotein; LDL, low-density lipoprotein; ROS, reactive oxygen species.

Alcohol can serve to both reduce chronic inflammation and increase vasodilation through the regulation of insulin resistance (Figure 1, Pathways: 1-14-54-69-70-89-100-85 and 1-14-54-69-72). This is beneficial to the RR for CHD through the regulation of these hallmarks. The effect of alcohol on acute insulin sensitivity is via a direct effect on fatty acid uptake in muscle tissue [22]. Therefore, a chronic increase in insulin sensitivity is due to reductions in adipose tissue and free fatty acid availability [22].

Moderate alcohol consumption has also been found to increase serum adiponectin levels [10,23]. Increases in plasma adiponectin concentrations can further increase insulin sensitivity by increasing muscle fat oxidation [24]. (Figure 1, Pathway: 1-49-19.)

Moderate alcohol consumption acts upon the liver and can therefore serve to directly increase the hepatic production and secretion of apolipoproteins and lipoprotein particles, increase triglyceride lipase concentrations, and decrease removal of circulating high density lipoprotein cholesterol [4]. Up-regulation of HDL or inhibition of LDL results in a reduction in the incidence of hypercholesterolaemia, which is a CHD hallmark. (Figure 1, Pathways: 1-12-33-51 and 1-10-31.)

Alcohol also reduces hyperglycaemia through the inhibition of hepatic gluconeogenesis, with a resulting reduction in plasma glucose levels. Reduced plasma glucose levels serve to decrease the incidence of hyperglycaemia and hyperinsulinaemia [25], which are both CHD hallmarks. (Figure 1, Pathway: 1-14-55.) However, it is acknowledged that the over-regulation of this specific pathway could also lead to hypoglycaemia in patients with heavy alcohol use [26].

It has also been noted that moderate alcohol use reduces fibrinogen levels, clotting factors, and platelet aggregation, which affects the CHD hallmark hypercoagulability. However, the precise mechanisms governing these reductions are not known [4]. (Figure 1, Pathway 1–49 and 1-49-75.)

From the above data it may be seen that the impact of ethanol consumption on the pathogenesis of CHD may highlight the potential methods of action in the lower relative risk of CHD associated with moderate alcohol consumption. Therefore, in order to further elucidate these effects we consider the impact of alcohol consumption on the biomarkers of CHD.

### Coronary heart disease biomarkers

The integrated model that was developed is a high-level conceptual model, from which the interconnectedness of CHD is immediately apparent (Figure 1). Therefore, in order to simplify the model, serological biomarkers (which quantifies the CHD pathways and which can be easily measured) were used to link the effect of moderate alcohol consumption to the corresponding risk of CHD [14].

Biomarkers can be used as indicators of an underlying disorder, such as systemic inflammation which is a known aggravating factor in the pathogenesis of CHD [27-29]. The measurement of specific biomarkers enables the prediction of the risk for CHD associated with said biomarker [30]. As it is also possible to accurately measure certain serum biomarker levels, they can be used as patient-specific links to pathogenetic or lifestyle factors (i.e. moderate alcohol consumption).

**Figure 2:**
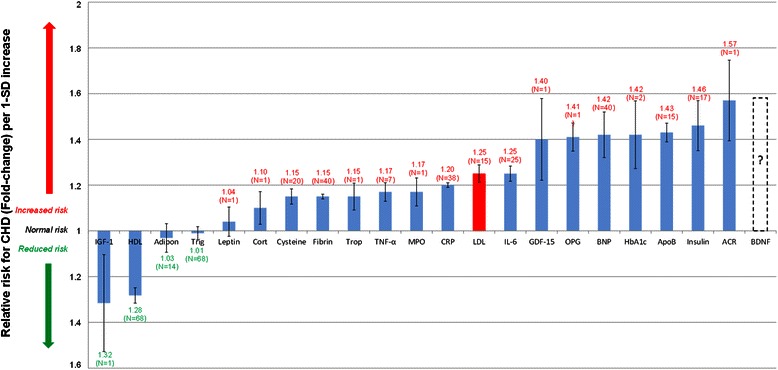
**Normalised relative risks (fold-change) of salient current biomarkers or of potential serological biomarkers for CHD.** From “How do high glycemic load diets influence coronary heart disease?” by Mathews MJ, Liebenberg L, Mathews EH. Nutr Metab.2015:12:6 [14] Increased IGF-1 and HDL levels are associated with a moderately decreased CHD risk. (IGF-1and HDL levels are significantly inversely correlated to relative risk for CHD.) *N* indicates number of trials; I, standard error; Adipo, adiponectin; HDL, high-density lipoprotein; BNP, B-type natriuretic peptide; ACR, albumin-to-creatinine ratio; GDF-15, growth-differentiation factor-15; Cysteine, Homocysteine; LDL, low-density lipoprotein; HbA_1c_, glycated haemoglobin A_1c_; Trop, troponins; Trigl, triglycerides; CRP, C-reactive protein; IL-6, interleukin-6; Fibrin, fibrinogen; Cort, cortisol; TNF-α, tumour necrosis factor-α; ApoB, apolipoprotein-B; IGF-1, insulin-like growth factor-1; MPO, myeloperoxidase; RANKL or OPG, osteoprotegerin; BDNF, brain-derived neurotrophic factor; HOMA, homeostasis model assessment.

A published study where all the important serum biomarkers were compared to show their relative importance regarding CHD risk prediction could not be found. This was therefore attempted in Table 3 with the corresponding results in Figure 2.

**Table 3:** **Salient serological and functional biomarkers of CHD, and prospective ones**

Biomarker (class and salient examples)	Prediction of CHD	Size of studies	Ref.
	Relative risk (95% CI)	*(N* = number of trials, *n* = number of patients)	
*Lipid-related markers:*			
HDL	0.78 (0.74-0.82)	(*N* = 68, *n* = 302 430)	[154]
Triglycerides	0.99 (0.94-1.05)	(*N* = 68, *n* = 302 430)	[154]
Leptin	1.04 (0.92-1.17)	(*n* = 1 832)	[155]
LDL	1.25 (1.18-1.33)	(*N* = 15, *n* = 233 455)	[156]
Non-HDL	1.34 (1.24-1.44)	(*N* = 15, *n* = 233 455)	[156]
ApoB	1.43 (1.35-1.51)	(*N* = 15, *n* = 233 455)	[156]
*Inflammation markers:*			
TNF-α	1.17 (1.09-1.25)	(*N* = 7, *n* = 6 107)	[157]
hsCRP	1.20 (1.18-1.22)	(*N* = 38, *n* = 166 596)	[158]
IL-6	1.25 (1.19-1.32)	(*N* = 25, *n* = 42 123)	[157]
GDF-15	1.40 (1.10-1.80)	(*n* = 1 740)	[159]
OPG	1.41 (1.33-1.57)	(*n* = 5 863)	[160]
*Marker of oxidative stress:*			
MPO	1.17 (1.06-1.30)	(*n* = 2 861)	[161]
*Marker of vascular function and neurohormonal activity:*			
Homocysteine	1.15 (1.09-1.22)	(*N* = 20, *n* = 22 652)	[162,163]
BNP	1.42 (1.24-1.63)	(*N* = 40*, n* = 87 474)	[164]
*Coagulation marker:*			
Fibrinogen	1.15 (1.13-1.17)	(*N* = 40*, n* = 185 892)	[158]
*Necrosis marker:*			
Troponins	1.15 (1.04-1.27)	(*n* = 3 265)	[20]
*Renal function marker:*			
Urinary ACR	1.57 (1.26-1.95)	(*n* = 626)	[165]
*Metabolic markers:*			
IGF-1	0.76 (0.56-1.04)	(*n* = 3 967)	[166]
Adiponectin	0.97 (0.86-1.09)	(*N* = 14, *n* =21 272)	[167]
Cortisol	1.10 (0.97-1.25)	(*n* = 2 512)	[168,169]
BDNF	**?**	***N/A***	[99,101,102]
HbA_1c_	1.42 (1.16-1.74)	(*N* = 2, *n* = 2 442)	[170]
Insulin resistance (HOMA)	1.46 (1.26-1.69)	(*N* = 17, *n* = 51 161)	[171]

From “How do high glycemic load diets influence coronary heart disease?” by Mathews MJ, Liebenberg L, Mathews EH. Nutr Metab. 2015:12:6 [14]. *n* denotes number of participants; *N*, number of trials; ?, currently unknown RR for CHD; HDL, high-density lipoprotein; BNP, B-type natriuretic peptide; ACR, albumin–to-creatinine ratio; GDF-15, growth-differentiation factor-15; LDL, low-density lipoprotein; HbA_1c_, glycated haemoglobin A_1c_; hsCRP, high-sensitivity C-reactive protein; IL-6, interleukin-6; TNF-α, tumour necrosis factor-α; ApoB, apolipoprotein-B; IGF-1, insulin-like growth factor-1; MPO, myeloperoxidase; RANKL or OPG, osteoprotegerin; BDNF, brain-derived neurotrophic factor; HOMA, homeostatic model assessment.

Table 3 presents the RR data from 294 cohort studies comprising 1 161 560 subjects. The results from these studies were interpreted and the averaged RR (with standard error (I) and study size (N)) was used to populate Figure 2. Figure 2 visually compares the RR of CHD associated with serological biomarkers per 1-standard deviation increase in said biomarker.

The comparative view of biomarker associated risks presented in Figure 2 elucidates the relative importance of various biomarkers of CHD. Using the array of biomarker risks and the integrated model developed in Figure 1 it is possible to display the interconnection of the pathogenesis of CHD and moderate consumption of alcohol. It may thus be possible to quantify the direct pathogenetic effects of moderate alcohol consumption on CHD through changes in biomarkers.

### Effects of moderate alcohol consumption

**Figure 3:**
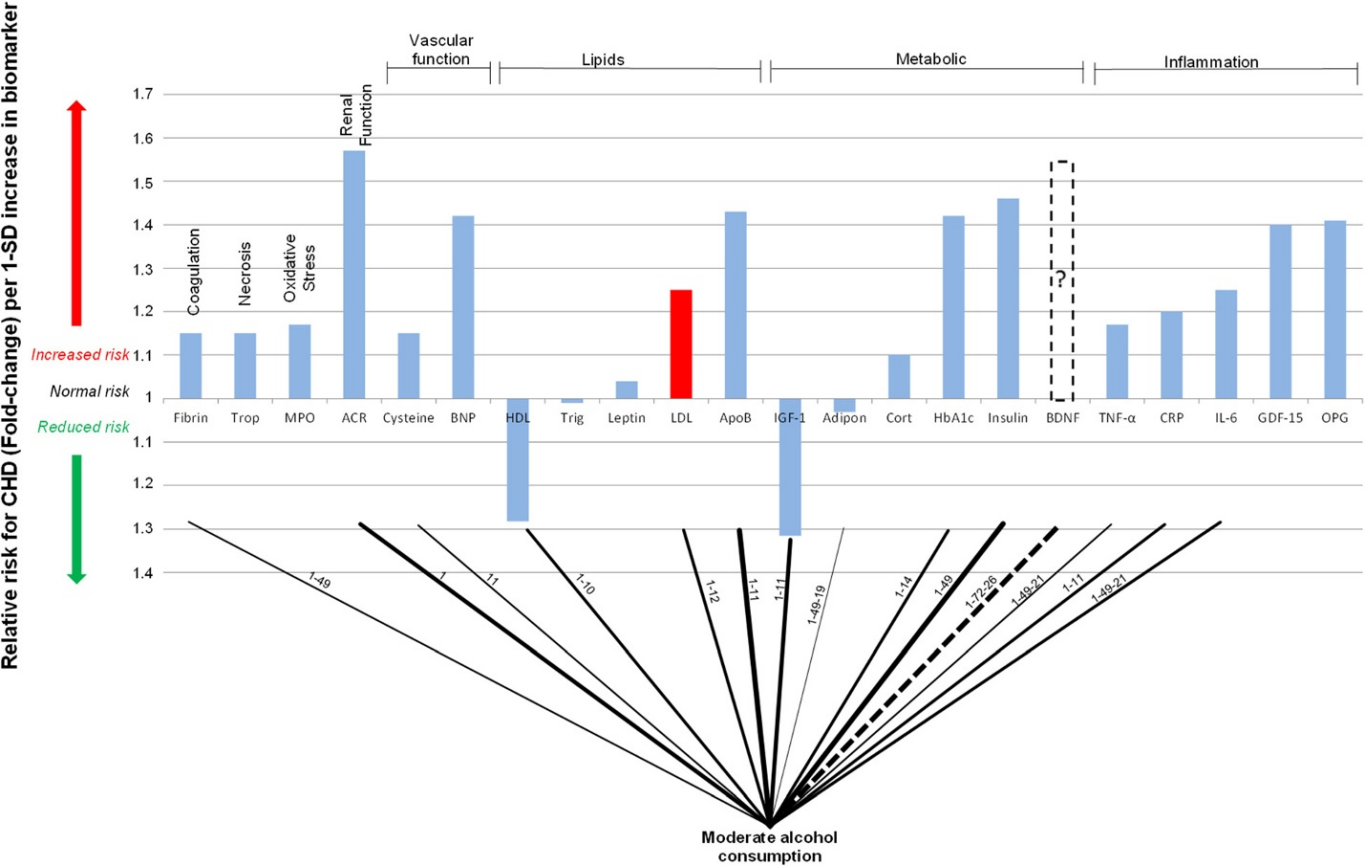
**Interconnection of relative risk effects of moderate alcohol consumption and serological biomarkers for CHD.** “ACR” denotes, albumin-to-creatinine ratio; Trop, troponins; Fibrin, fibrinogen; MPO, myeloperoxidase; BNP, B-type natriuretic peptide; Cysteine, Homocysteine; HDL, high-density lipoprotein; LDL, low-density lipoprotein; Trigl, triglycerides; ApoB, Apolipoprotein-B; Adipon, adiponectin; HbA_1c_, glycated haemoglobin A_1c_; Cort, cortisol; IGF-1, insulin-like growth factor-1; BDNF, brain-derived neurotrophic factor; GDF-15, growth-differentiation factor-15; CRP, C-reactive protein; IL-6, interleukin-6; TNF-α, tumour necrosis factor-α; RANKL or OPG, osteoprotegerin.

By combining the array of biomarker risks and the pathogenetic pathways elucidated from Figure 2 a connection graph which displays the pathogenetic connections between moderate alcohol consumption and CHD biomarker risk was developed. The pathogenetic pathways (from Figure 1), which are elucidated by the associated biomarker, are superimposed on the connecting lines in Figure 3. Increasing line thickness indicates a connection with greater pathogenetic effect (as quantified by biomarker risk prediction of CHD). For example, the risk of CHD is relatively low when considering adiponectin, thus the connection line between moderate alcohol consumption and adiponectin is thin.

From the connection graph in Figure 3 it is clear that moderate alcohol consumption is widely connected to the biomarkers associated with CHD risk. It is apparent that there are multiple connections between moderate alcohol consumption and the metabolic function biomarkers, the inflammation biomarkers, as well as a connection to the coagulation biomarker, fibrinogen.

The connections between alcohol consumption and inflammation are evident from Figure 3. The anti-inflammatory effect associated with moderate alcohol consumption could explain some of the lower CHD risk. Imhof and co-workers however found that excessive and no consumption of alcohol, led to higher serum levels of CRP compared to moderate alcohol consumption [31,32]. This indicates that excessive alcohol consumption can increase inflammation and a patient’s risk for CHD, and therefore emphasises that care needs to be taken when contemplating moderate alcohol use as a lifestyle factor for CHD prevention [33].

Moderate alcohol intake has also been connected to an increase in adiponectin levels [10,13,23], which can lead to a reduction in adipose tissue; this in turn can increase insulin sensitivity and decrease inflammation [34]. Thus, some of the reduction in inflammation, and the concomitant decrease in CHD risk, may be accounted for by increased serum levels of adiponectin.

Various cohort studies have also observed that fibrinogen serum reduce after moderate alcohol consumption in [4,11,12,35]. This leads to a reduction in hypercoagulability, which would reduce the risk for CHD events.

Overall, the increase in HDL is thought to account for 50% of the lower CHD risk observed in those consuming alcohol in moderation [36]. The remaining lowered CHD risk is thought to be due to the anti-thrombotic effects of decreased fibrinogen serum levels [12] and increased serum levels of adiponectin [10,23].

However, from the connection graph in Figure 3 it is deemed plausible that a portion of the lower risk for CHD associated with moderate alcohol consumption may also be due to an anti-inflammatory effect, independent of increased adiponectin levels [32].

Although the numerical values of RR presented here are based on large, clustered clinical trials, and thus give a good idea of *average* effects, it is acknowledged that individual patients will have very specific CHD profiles. However, Figure 1 is still relevant to everyone and should thus provide general insight. Therefore, Figure 1 could *inter alia* reveal pathways still available for further biomarker and drug discovery.

## Discussion

It is well documented that the lowered CHD risk associated with moderate alcohol consumption is independent of beverage type [12,32,35]. This underscores the hypothesis that the lower risk of CHD associated with consuming alcohol in moderation is due to the ethanol content consumed and not the non-alcoholic components of the beverages [35].

It has however been suggested in one study that the lower risk for CHD associated with moderate alcohol consumption may be entirely due to higher socioeconomic status which is more prevalent with persons who consume moderate amounts of alcohol [37]. However, the results with regards to changes in serological biomarkers, as shown in Figure 3, would indicate that alcohol consumption does attributes some positive action on the pathogenesis of CHD.

The majority of studies show that moderate alcohol consumption conforms to a lower risk for CHD [4-8]. It therefore appears to validate the observation that moderate alcohol use could be a suitable lifestyle factor to consider for the prevention of CHD. However, the use of alcohol as a preventative treatment is complex due to both the potential adverse effects associated with alcohol, and as alcohol abuse contributes greatly to preventable deaths in the United States [33].

Additionally, it has been found that teetotallers and drinkers of fewer than one drink a month have a greater risk for fatal CHD than moderate and even heavy drinkers [5]. However, heavy drinkers have an increased risk of myocardial infarction [5].

Excessive alcohol consumption, more than 30 g per day, has also been associated with hypertension [38], declining ejection fraction [39], progressive left ventricular hypertrophy [40], increased risk of stroke [41], dementia [42] and overall mortality [43]. Thus, it is extremely important that alcohol use be constrained to moderate consumption levels, of 20–30 grams of ethanol per day for men and 10–15 grams of ethanol per day for women, in order to gain a potential benefit from its use.

It is further acknowledged that moderate alcohol consumption may not be possible due to religious or personal reasons. In addition, caution is advised in recommending moderate alcohol use to patients who had not previously consumed alcohol regularly, or at all [40]. Serious consideration should also be taken with patients that have a family history of alcohol abuse, addiction or depression. Furthermore, the lower risk for CHD in moderate alcohol consumers has been found to be more evident in middle-aged (50–59 years) and older adults (≥60 years) compared to younger adults (≤50 years) [3].

The current data regarding the consumption of alcohol in CHD risk reduction is based largely on observational studies [9]. However, based on the wide connection and effect of alcohol on the biomarkers, as shown in Figure 3, and pathogenetic pathways of CHD (Figures 1 and 3) it seems plausible that moderate alcohol consumption may prove a causal factor in CHD risk reduction. Thus it may be possible that the consumption of alcohol in a moderate dosage of 20–30 grams for men and 10–15 grams for women may prove beneficial to overall CHD risk.

## Conclusions

Moderate alcohol consumption is associated with a lower risk of CHD. This lower risk has been observed independent of the beverage type consumed. A high-level conceptual model has been developed which links moderate alcohol consumption, and the pathogenesis and hallmarks of CHD.

This shows the positive effect of moderate alcohol consumption on certain important aspects of the pathogenesis of CHD and may explain why moderate alcohol consumption is associated with lower CHD risk. It is now clear at a glance that moderate alcohol consumption increases HDL-cholesterol, insulin sensitivity and adiponectin levels while decreasing inflammation, all of which have positive effects on the risk for CHD.

The integrated high level CHD model provides a summary of evidence for a causal relationship between CHD risk and moderate alcohol consumption.

## References

[1] Mathers CD, Boerma T, Fat DM (2009). Global and regional causes of death. Br Med Bull.

[2] Gaziano JM, Buring JE, Breslow JL, Goldhaber SZ, Rosner B, VanDenburgh M (1993). Moderate alcohol intake, increased levels of high-density lipoprotein and its subfractions, and decreased risk of myocardial infarction. N Engl J Med.

[3] Hvidtfeldt UA, Tolstrup JS, Jakobsen MU, Heitmann BL, Grønbæk M, O'Reilly E (2010). Alcohol intake and risk of coronary heart disease in younger, middle-aged, and older adults. Circulation.

[4] Rimm EB, Williams P, Fosher K, Criqui M, Stampfer MJ (1999). Moderate alcohol intake and lower risk of coronary heart disease: meta-analysis of effects on lipids and haemostatic factors. BMJ.

[5] Jackson R, Scragg R, Beaglehole R (1991). Alcohol consumption and risk of coronary heart disease. BMJ.

[6] Marmot M (1984). Alcohol and coronary heart disease. Int J Epidemiol.

[7] Ikehara S, Iso H, Toyoshima H, Date C, Yamamoto A, Kikuchi S (2008). Alcohol consumption and mortality from stroke and coronary heart disease among Japanese men and women: The Japan Collaborative Cohort study. Stroke.

[8] Arriola L, Martinez-Camblor P, Larrañaga N, Basterretxea M, Amiano P, Moreno-Iribas C (2010). Alcohol intake and the risk of coronary heart disease in the Spanish EPIC cohort study. Heart.

[9] Ronksley PE, Brien SE, Turner BJ, Mukamal KJ, Ghali WA (2011). Association of alcohol consumption with selected cardiovascular disease outcomes: a systematic review and meta-analysis. BMJ.

[10] Pischon T, Girman CJ, Rifai N, Hotamisligil GS, Rimm EB (2005). Association between dietary factors and plasma adiponectin concentrations in men. Am J Clin Nutr.

[11] Sierksma A, Van Der Gaag M, Kluft C, Hendriks H (2002). Moderate alcohol consumption reduces plasma C-reactive protein and fibrinogen levels; a randomized, diet-controlled intervention study. Eur J Clin Nutr.

[12] Brien SE, Ronksley PE, Turner BJ, Mukamal KJ, Ghali WA (2011). Effect of alcohol consumption on biological markers associated with risk of coronary heart disease: systematic review and meta-analysis of interventional studies. BMJ.

[13] Sierksma A, Patel H, Ouchi N, Kihara S, Funahashi T, Heine RJ (2004). Effect of moderate alcohol consumption on adiponectin, tumor necrosis factor-α, and insulin sensitivity. Diabetes Care.

[14] Mathews M, Liebenberg L, Mathews E (2015). How do high glycemic load diets influence coronary heart disease?. Nutr Metab.

[15] Kavvoura FK, Liberopoulos G, Ioannidis JP (2007). Selection in reported epidemiological risks: an empirical assessment. PLoS Med.

[16] Zou G (2004). A modified poisson regression approach to prospective studies with binary data. Am J Epidemiol.

[17] Lusis AJ, Weiss JN (2010). Cardiovascular networks systems-based approaches to cardiovascular disease. Circulation.

[18] Lusis AJ, Attie AD, Reue K (2008). Metabolic syndrome: from epidemiology to systems biology. Nat Rev Genet.

[19] Libby P, Ridker PM, Hansson GK (2011). Progress and challenges in translating the biology of atherosclerosis. Nature.

[20] Wang TJ, Wollert KC, Larson MG, Coglianese E, McCabe EL, Cheng S (2012). Prognostic utility of novel biomarkers of cardiovascular stress: the Framingham Heart Study. Circulation.

[21] Diez D, Wheelock ÅM, Goto S, Haeggström JZ, Paulsson-Berne G, Hansson GK (2010). The use of network analyses for elucidating mechanisms in cardiovascular disease. Mol Biosyst.

[22] Greenfield JR, Samaras K, Jenkins AB, Kelly PJ, Spector TD, Campbell LV (2003). Moderate alcohol consumption, estrogen replacement therapy, and physical activity are associated with increased insulin sensitivity is abdominal adiposity the mediator?. Diabetes Care.

[23] Beulens JW, van Loon LJ, Kok FJ, Pelsers M, Bobbert T, Spranger J (2007). The effect of moderate alcohol consumption on adiponectin oligomers and muscle oxidative capacity: a human intervention study. Diabetologia.

[24] Yamauchi T, Kamon J, Waki H, Terauchi Y, Kubota N, Hara K (2001). The fat-derived hormone adiponectin reverses insulin resistance associated with both lipoatrophy and obesity. Nat Med.

[25] Siler SQ, Neese RA, Christiansen MP, Hellerstein MK (1998). The inhibition of gluconeogenesis following alcohol in humans. Am J Physiol Endocrinol Metab.

[26] Krebs H, Freedland R, Hems R, Stubbs M (1969). Inhibition of hepatic gluconeogenesis by ethanol. Biochem J.

[27] Libby P (2002). Atherosclerosis in inflammation. Nature.

[28] Packard RR, Libby P (2008). Inflammation in atherosclerosis: from vascular biology to biomarker discovery and risk prediction. Clin Chem.

[29] Becker AE, de Boer OJ, van der Wal AC (2001). The role of inflammation and infection in coronary artery disease. Annu Rev Med.

[30] Vasan RS (2006). Biomarkers of cardiovascular disease molecular basis and practical considerations. Circulation.

[31] Imhof A, Froehlich M, Brenner H, Boeing H, Pepys MB, Koenig W (2001). Effect of alcohol consumption on systemic markers of inflammation. The Lancet.

[32] Imhof A, Woodward M, Doering A, Helbecque N, Loewel H, Amouyel P (2004). Overall alcohol intake, beer, wine, and systemic markers of inflammation in western Europe: results from three MONICA samples (Augsburg, Glasgow, Lille). Eur Heart J.

[33] O’Keefe JH, Bybee KA, Lavie CJ (2007). Alcohol and Cardiovascular HealthThe Razor-Sharp Double-Edged Sword. J Am Coll Cardiol.

[34] Oliveira CS, Giuffrida F, Crispim F, Saddi-Rosa P, Reis AF (2011). ADIPOQ and adiponectin: the common ground of hyperglycemia and coronary artery disease?. Arq Bras Endocrinol Metabol.

[35] Mukamal KJ, Jensen MK, Grønbæk M, Stampfer MJ, Manson JE, Pischon T (2005). Drinking frequency, mediating biomarkers, and risk of myocardial infarction in women and men. Circulation.

[36] Klatsky A (2001). Commentary: Could abstinence from alcohol be hazardous to your health?. Int J Epidemiol.

[37] Hart CL, Davey Smith G, Hole DJ, Hawthorne VM (1999). Alcohol consumption and mortality from all causes, coronary heart disease, and stroke: results from a prospective cohort study of Scottish men with 21 years of follow up. BMJ.

[38] Beilin LJ, Puddey IB (2006). Alcohol and Hypertension An Update. Hypertension.

[39] De Leiris J, de Lorgeril M, Boucher F (2006). Ethanol and cardiac function. Am J Physiol Heart Circ Physiol.

[40] Lucas DL, Brown RA, Wassef M, Giles TD (2005). Alcohol and the Cardiovascular SystemResearch Challenges and Opportunities. J Am Coll Cardiol.

[41] Klatsky AL (2005). Editorial Comment—Alcohol and Stroke An Epidemiological Labyrinth. Stroke.

[42] Mukamal KJ, Kuller LH, Fitzpatrick AL, Longstreth W, Mittleman MA, Siscovick DS (2003). Prospective study of alcohol consumption and risk of dementia in older adults. JAMA.

[43] Mukamal KJ, Maclure M, Muller JE, Mittleman MA (2005). Binge drinking and mortality after acute myocardial infarction. Circulation.

[44] Bollen M, Keppens S, Stalmans W (1998). Specific features of glycogen metabolism in the liver. Biochem J.

[45] Uchiki T, Weikel KA, Jiao W, Shang F, Caceres A, Pawlak D (2012). Glycation‐altered proteolysis as a pathobiologic mechanism that links dietary glycemic index, aging, and age‐related disease (in nondiabetics). Aging Cell.

[46] Waqar AB, Koike T, Yu Y, Inoue T, Aoki T, Liu E (2010). High-fat diet without excess calories induces metabolic disorders and enhances atherosclerosis in rabbits. Atherosclerosis.

[47] Wang Z, Klipfell E, Bennett BJ, Koeth R, Levison BS, DuGar B (2011). Gut flora metabolism of phosphatidylcholine promotes cardiovascular disease. Nature.

[48] Strowig T, Henao-Mejia J, Elinav E, Flavell R (2012). Inflammasomes in health and disease. Nature.

[49] Shanahan F (2012). The gut microbiota-a clinical perspective on lessons learned. Nat Rev Gastroentero.

[50] Golbidi S, Laher I (2012). Exercise and the cardiovascular system. Cardiol Res Pract.

[51] Macera CA, Hootman JM, Sniezek JE (2003). Major public health benefits of physical activity. Arthritis Care Res.

[52] Thompson PD (2003). Exercise and physical activity in the prevention and treatment of atherosclerotic cardiovascular disease. Arterioscler Thromb Vasc Biol.

[53] Badrick E, Kirschbaum C, Kumari M (2007). The relationship between smoking status and cortisol secretion. J Clin Endocrinol Metab.

[54] Reaven G, Tsao PS (2003). Insulin resistance and compensatory hyperinsulinemia. The key player between cigarette smoking and cardiovascular disease?. J Am Coll Cardiol.

[55] Efstathiou SP, Skeva II, Dimas C, Panagiotou A, Parisi K, Tzanoumis L (2009). Smoking cessation increases serum adiponectin levels in an apparently healthy Greek population. Atherosclerosis.

[56] Granados-Principal S, El-Azem N, Quiles JL, Perez-Lopez P, Gonzalez A, Ramirez-Tortosa M, Gasparyan AY (2012). Relationship between cardiovascular risk factors and periodontal disease: current knowledge. Cardiovascular Risk Factors.

[57] Meurman JH, Sanz M, Janket S-J (2004). Oral health, atherosclerosis, and cardiovascular disease. Crit Rev Oral Biol Med.

[58] Fisher MA, Borgnakke WS, Taylor GW (2010). Periodontal disease as a risk marker in coronary heart disease and chronic kidney disease. Curr Opin Nephrol Hypertens.

[59] Walker BR (2007). Glucocorticoids and cardiovascular disease. Eur J Endocrinol.

[60] Costa R, Sanches A, Cunha TS, Moura MJCS, Tanno AP, Casarini DE (2011). Dyslipidemia induced by stress. Dyslipidemia - From Prevention to Treatment.

[61] McEwen BS (2008). Central effects of stress hormones in health and disease: understanding the protective and damaging effects of stress and stress mediators. Eur J Pharmacol.

[62] Musselman DL, Evans DL, Nemeroff CB (1998). The relationship of depression to cardiovascular disease: epidemiology, biology, and treatment. Arch Gen Psychiatry.

[63] Celano CM, Huffman JC (2011). Depression and cardiac disease: a review. Cardiol Rev.

[64] von Känel R (2012). Psychosocial stress and cardiovascular risk: current opinion. Swiss Med Wkly.

[65] Sher Y, Lolak S, Maldonado JR (2010). The impact of depression in heart disease. Curr Psychiatry Rep.

[66] Everson-Rose SA, Lewis TT, Karavolos K, Dugan SA, Wesley D, Powell LH (2009). Depressive symptoms and increased visceral fat in middle-aged women. Psychosom Med.

[67] Weber-Hamann B, Werner M, Hentschel F, Bindeballe N, Lederbogen F, Deuschle M (2006). Metabolic changes in elderly patients with major depression: evidence for increased accumulation of visceral fat at follow-up. Psychoneuroendocrino.

[68] Spiegel K, Tasali E, Leproult R, Van Cauter E (2009). Effects of poor and short sleep on glucose metabolism and obesity risk. Nat Rev Endocrinol.

[69] Spiegel K, Knutson K, Leproult R, Tasali E, Van Cauter E (2005). Sleep loss: a novel risk factor for insulin resistance and Type 2 diabetes. J Appl Physiol.

[70] Knutson KL, Spiegel K, Penev P, Van Cauter E (2007). The metabolic consequences of sleep deprivation. Sleep Med Rev.

[71] Shamsuzzaman AS, Gersh BJ, Somers VK (2003). Obstructive sleep apneaimplications for cardiac and vascular disease. JAMA.

[72] Jackson SP (2011). Arterial thrombosis-insidious, unpredictable and deadly. Nat Med.

[73] Vykoukal D, Davies MG (2011). Vascular biology of metabolic syndrome. J Vasc Surg.

[74] Heinecke JW (2012). The not-so-simple HDL story: a new era for quantifying HDL and cardiovascular risk?. Nat Med.

[75] Rader DJ, Tall AR (2012). The not-so-simple HDL story: Is it time to revise the HDL cholesterol hypothesis?. Nat Med.

[76] Aronson D, Rayfield EJ (2002). How hyperglycemia promotes atherosclerosis: molecular mechanisms. Cardiovasc Diabetol.

[77] Beckman JA, Creager MA, Libby P (2002). Diabetes and atherosclerosis: epidemiology, pathophysiology, and management. JAMA.

[78] Rydén L, Standl E, Bartnik M, Van den Berghe G, Betteridge J, De Boer M-J (2007). Guidelines on diabetes, pre-diabetes, and cardiovascular diseases: executive summary The Task Force on Diabetes and Cardiovascular Diseases of the European Society of Cardiology (ESC) and of the European Association for the Study of Diabetes (EASD). Eur Heart J.

[79] Stumvoll M, Goldstein BJ, van Haeften TW (2005). Type 2 diabetes: principles of pathogenesis and therapy. Lancet.

[80] Gerstein HC, Miller ME, Genuth S, Ismail-Beigi F, Buse JB, Goff DC (2011). Long-term effects of intensive glucose lowering on cardiovascular outcomes. N Engl J Med.

[81] Khardori R, Nguyen DD (2012). Glucose control and cardiovascular outcomes: reorienting approach. Front Endocrinol (Lausanne).

[82] Gorgojo Martínez JJ (2011). Glucocentricity or adipocentricity: a critical view of consensus and clinical guidelines for the treatment of type 2 diabetes mellitus. Endocrinol Nutr.

[83] Zoungas S, Chalmers J, Ninomiya T, Li Q, Cooper M, Colagiuri S (2012). Association of HbA1c levels with vascular complications and death in patients with type 2 diabetes: evidence of glycaemic thresholds. Diabetologia.

[84] Leibundgut G, Arai K, Orsoni A, Yin H, Scipione C, Miller ER (2012). Oxidized phospholipids are present on plasminogen, affect fibrinolysis, and increase following acute myocardial infarction. J Am Coll Cardiol.

[85] Tsimikas S, Hall JL (2012). Lipoprotein (a) as a potential causal genetic risk factor of cardiovascular disease: a rationale for increased efforts to understand its pathophysiology and develop targeted therapies. J Am Coll Cardiol.

[86] Cypess AM, Lehman S, Williams G, Tal I, Rodman D, Goldfine AB (2009). Identification and importance of brown adipose tissue in adult humans. N Engl J Med.

[87] Levitan EB, Cook NR, Stampfer MJ, Ridker PM, Rexrode KM, Buring JE (2008). Dietary glycemic index, dietary glycemic load, blood lipids, and C-reactive protein. Metabolism.

[88] Takefuji S, Yatsuya H, Tamakoshi K, Otsuka R, Wada K, Matsushita K (2007). Smoking status and adiponectin in healthy Japanese men and women. Prev Med.

[89] Tsai J-S, Guo F-R, Chen S-C, Lue B-H, Chiu T-Y, Chen C-Y (2011). Smokers show reduced circulating adiponectin levels and adiponectin mRNA expression in peripheral blood mononuclear cells. Atherosclerosis.

[90] Epstein FH, Ross R (1999). Atherosclerosis—an inflammatory disease. N Engl J Med.

[91] Trepels T, Zeiher AM, Fichtlscherer S (2006). The endothelium and inflammation. Endothelium.

[92] Paquette DW, Brodala N, Nichols TC. Cardiovascular disease, inflammation, and periodontal infection. Periodontol 2000. 2007;44:113–26. 10.1111/j.1600-0757.2006.00196.x 17474929

[93] Persson GR, Persson RE (2008). Cardiovascular disease and periodontitis: an update on the associations and risk. J Clin Periodontol.

[94] Kebschull M, Demmer R, Papapanou P (2010). “Gum bug, leave my heart alone!”-epidemiologic and mechanistic evidence linking periodontal infections and atherosclerosis. J Dent Res.

[95] Machuca G, Segura-Egea JJ, Jiménez-Beato G, Lacalle JR, Bullón P (2012). Clinical indicators of periodontal disease in patients with coronary heart disease: A 10 years longitudinal study. Med Oral Patol Oral Cir Bucal.

[96] Li X, Kolltveit KM, Tronstad L, Olsen I (2000). Systemic diseases caused by oral infection. Clin Microbiol Rev.

[97] Krishnan V, Nestler EJ (2008). The molecular neurobiology of depression. Nature.

[98] Raison CL, Capuron L, Miller AH (2006). Cytokines sing the blues: inflammation and the pathogenesis of depression. Trends Immunol.

[99] Noble EE, Billington CJ, Kotz CM, Wang C (2011). The lighter side of BDNF. Am J Physiol Regul Integr Comp Physiol.

[100] Feder A, Nestler EJ, Charney DS (2009). Psychobiology and molecular genetics of resilience. Nat Rev Neurosci.

[101] Karatsoreos IN, McEwen BS (2011). Psychobiological allostasis: resistance, resilience and vulnerability. Trends Cogn Sci.

[102] Calabrese F, Molteni R, Racagni G, Riva MA (2009). Neuronal plasticity: a link between stress and mood disorders. Psychoneuroendocrino.

[103] Liebenberg L, Mathews EH (2012). A practical quantification of blood glucose production due to high-level chronic stress. Stress Health.

[104] Kubzansky LD, Kawachi I, Spiro A, Weiss ST, Vokonas PS, Sparrow D (1997). Is worrying bad for your heart? A prospective study of worry and coronary heart disease in the Normative Aging Study. Circulation.

[105] Einvik G, Ekeberg Ø, Klemsdal TO, Sandvik L, Hjerkinn EM (2009). Physical distress is associated with cardiovascular events in a high risk population of elderly men. BMC Cardiovasc Disord.

[106] Vogelzangs N, Seldenrijk A, Beekman AT, van Hout HP, de Jonge P, Penninx BW (2010). Cardiovascular disease in persons with depressive and anxiety disorders. J Affect Disord.

[107] Dungan KM, Braithwaite SS, Preiser J-C (2009). Stress hyperglycaemia. Lancet.

[108] Dallman MF, Akana SF, Laugero KD, Gomez F, Manalo S, Bell M (2003). A spoonful of sugar: feedback signals of energy stores and corticosterone regulate responses to chronic stress. Physiol Behav.

[109] Weissman C (1990). The metabolic response to stress: an overview and update. Anesthesiology.

[110] Kohler M, Stradling JR (2010). Mechanisms of vascular damage in obstructive sleep apnea. Nat Rev Cardiol.

[111] Levy P, Bonsignore M, Eckel J (2009). Sleep, sleep-disordered breathing and metabolic consequences. Eur Respir J.

[112] Punjabi NM, Polotsky VY (2005). Disorders of glucose metabolism in sleep apnea. J Appl Physiol.

[113] Besedovsky L, Lange T, Born J (2012). Sleep and immune function. Pflügers Arch Eur J Physiol.

[114] Ip MS, Lam B, Ng MM, Lam WK, Tsang KW, Lam KS (2002). Obstructive sleep apnea is independently associated with insulin resistance. Am J Respir Crit Care Med.

[115] Bornfeldt KE, Tabas I (2011). Insulin resistance, hyperglycemia, and atherosclerosis. Cell.

[116] Muniyappa R, Montagnani M, Koh KK, Quon MJ (2007). Cardiovascular actions of insulin. Endocr Rev.

[117] Nigro J, Osman N, Dart AM, Little PJ (2006). Insulin resistance and atherosclerosis. Endocr Rev.

[118] Saltiel AR, Kahn CR (2001). Insulin signalling and the regulation of glucose and lipid metabolism. Nature.

[119] Samuel VT, Shulman GI (2012). Mechanisms for insulin resistance: common threads and missing links. Cell.

[120] Stocker R, Keaney JF (2004). Role of oxidative modifications in atherosclerosis. Physiol Rev.

[121] Van Gaal LF, Mertens IL, Christophe E (2006). Mechanisms linking obesity with cardiovascular disease. Nature.

[122] Vettore M, Leao A (2003). Monteiro Da Silva A, Quintanilha R. Lamarca G The relationship of stress and anxiety with chronic periodontitis J Clin Periodontol.

[123] Ng SK, Keung LW (2006). A community study on the relationship between stress, coping, affective dispositions and periodontal attachment loss. Community Dent Oral Epidemiol.

[124] Michel T, Vanhoutte PM (2010). Cellular signaling and NO production. Pflügers Arch Eur J Physiol.

[125] Biondi-Zoccai GG, Abbate A, Liuzzo G, Biasucci LM (2003). Atherothrombosis, inflammation, and diabetes. J Am Coll Cardiol.

[126] Schadt EE (2009). Molecular networks as sensors and drivers of common human diseases. Nature.

[127] Kitano H, Oda K, Kimura T, Matsuoka Y, Csete M, Doyle J (2004). Metabolic syndrome and robustness tradeoffs. Diabetes.

[128] Wang JC, Bennett M (2012). Aging and atherosclerosis mechanisms, functional consequences, and potential therapeutics for cellular senescence. Circ Res.

[129] Eckel RH, Grundy SM, Zimmet PZ (2005). The metabolic syndrome. Lancet.

[130] Mazzone T, Chait A, Plutzky J (2008). Cardiovascular disease risk in type 2 diabetes mellitus: insights from mechanistic studies. Lancet.

[131] Libby P, DiCarli M, Weissleder R (2010). The vascular biology of atherosclerosis and imaging targets. J Nucl Med.

[132] Mougios V (2006). Exercise biochemistry.

[133] Wykrzykowska J, Lehman S, Williams G, Parker JA, Palmer MR, Varkey S (2009). Imaging of inflamed and vulnerable plaque in coronary arteries with 18F-FDG PET/CT in patients with suppression of myocardial uptake using a low-carbohydrate, high-fat preparation. J Nucl Med.

[134] Sanz J, Fayad ZA (2008). Imaging of atherosclerotic cardiovascular disease. Nature.

[135] Rudd JH, Hyafil F, Fayad ZA (2009). Inflammation imaging in atherosclerosis. Arterioscler Thromb Vasc Biol.

[136] Christen T, Sheikine Y, Rocha VZ, Hurwitz S, Goldfine AB, Di Carli M (2010). Increased glucose uptake in visceral versus subcutaneous adipose tissue revealed by PET imaging. JACC Cardiovasc Imaging.

[137] Du XL, Edelstein D, Dimmeler S, Ju Q, Sui C, Brownlee M (2001). Hyperglycemia inhibits endothelial nitric oxide synthase activity by posttranslational modification at the Akt site. J Clin Invest.

[138] Vanhoutte P, Shimokawa H, Tang E, Feletou M (2009). Endothelial dysfunction and vascular disease. Acta Physiol Scand.

[139] Rader DJ, Daugherty A (2008). Translating molecular discoveries into new therapies for atherosclerosis. Nature.

[140] Pountos I, Georgouli T, Bird H, Giannoudis PV (2011). Nonsteroidal anti-inflammatory drugs: prostaglandins, indications, and side effects. Int J Infereron Cytokine Mediator Res.

[141] Chan D, Ng LL (2010). Biomarkers in acute myocardial infarction. BMC Med.

[142] Singh V, Tiwari RL, Dikshit M, Barthwal MK (2009). Models to study atherosclerosis: a mechanistic insight. Curr Vasc Pharmacol.

[143] Koo JW, Russo SJ, Ferguson D, Nestler EJ, Duman RS (2010). Nuclear factor-κB is a critical mediator of stress-impaired neurogenesis and depressive behavior. Proc Natl Acad Sci U S A.

[144] Bierhaus A, Wolf J, Andrassy M, Rohleder N, Humpert PM, Petrov D (2003). A mechanism converting psychosocial stress into mononuclear cell activation. Proc Natl Acad Sci U S A.

[145] von Känel R, Mills PJ, Fainman C, Dimsdale JE (2001). Effects of psychological stress and psychiatric disorders on blood coagulation and fibrinolysis: a biobehavioral pathway to coronary artery disease?. Psychosom Med.

[146] Haroon E, Raison CL, Miller AH (2012). Psychoneuroimmunology meets neuropsychopharmacology: translational implications of the impact of inflammation on behavior. Neuropsychopharmacol.

[147] von der Thüsen JH, Borensztajn KS, Moimas S, van Heiningen S, Teeling P, van Berkel TJ (2011). IGF-1 has plaque-stabilizing effects in atherosclerosis by altering vascular smooth muscle cell phenotype. Am J Pathol.

[148] Shai S-Y, Sukhanov S, Higashi Y, Vaughn C, Rosen CJ, Delafontaine P (2011). Low circulating insulin-like growth factor I increases atherosclerosis in ApoE-deficient mice. Am J Physiol Heart Circ Physiol.

[149] Ruidavets J, Luc G, Machez E, Genoux A, Kee F, Arveiler D (2011). Effects of insulin-like growth factor 1 in preventing acute coronary syndromes: The PRIME study. Atherosclerosis.

[150] Higashi Y, Sukhanov S, Anwar A, Shai S-Y, Delafontaine P (2012). Aging, atherosclerosis, and IGF-1. J Gerontol A Biol Sci Med Sci.

[151] Krishnadas R, Cavanagh J (2012). Depression: an inflammatory illness?. J Neurol Neurosurg Psychiatry.

[152] Gardner A, Boles RG (2011). Beyond the serotonin hypothesis: mitochondria, inflammation and neurodegeneration in major depression and affective spectrum disorders. Prog Neuropsychopharmacol Biol Psychiatry.

[153] Mokuda O, Tanaka H, Hayashi T, Ooka H, Okazaki R, Sakamoto Y (2004). Ethanol stimulates glycogenolysis and inhibits both glycogenesis via gluconeogenesis and from exogenous glucose in perfused rat liver. Ann Nutr Metab.

[154] Di Angelantonio E, Sarwar N, Perry P, Kaptoge S, Ray KK, Thompson A (2009). Major lipids, apolipoproteins, and risk of vascular disease. JAMA.

[155] Luc G, Empana J, Morange P, Juhan-Vague I, Arveiler D, Ferrieres J (2009). Adipocytokines and the risk of coronary heart disease in healthy middle aged men: the PRIME Study. Int J Obes.

[156] Sniderman AD, Williams K, Contois JH, Monroe HM, McQueen MJ, de Graaf J (2011). A meta-analysis of low-density lipoprotein cholesterol, non-high-density lipoprotein cholesterol, and apolipoprotein B as markers of cardiovascular risk. Circ Cardiovasc Qual Outcomes.

[157] Kaptoge S, Seshasai SRK, Gao P, Freitag DF, Butterworth AS, Borglykke A (2014). Inflammatory cytokines and risk of coronary heart disease: new prospective study and updated meta-analysis. Eur Heart J.

[158] Kaptoge S, Di Angelantonio E, Pennells L, Wood AM, White IR, Gao P (2012). C-reactive protein, fibrinogen, and cardiovascular disease prediction. N Engl J Med.

[159] Daniels LB, Clopton P, Laughlin GA, Maisel AS, Barrett-Connor E (2011). Growth-Differentiation Factor-15 Is a Robust, Independent Predictor of 11-Year Mortality Risk in Community-Dwelling Older Adults The Rancho Bernardo Study. Circulation.

[160] Mogelvang R, Pedersen SH, Flyvbjerg A, Bjerre M, Iversen AZ, Galatius S (2012). Comparison of osteoprotegerin to traditional atherosclerotic risk factors and high-sensitivity C-reactive protein for diagnosis of atherosclerosis. Am J Cardiol.

[161] Rana JS, Arsenault BJ, Després J-P, Côté M, Talmud PJ, Ninio E (2009). Inflammatory biomarkers, physical activity, waist circumference, and risk of future coronary heart disease in healthy men and women. Eur Heart J.

[162] Humphrey LL, Fu R, Rogers K, Freeman M, Helfand M (2008). Homocysteine level and coronary heart disease incidence: a systematic review and meta-analysis. Mayo Clin Proc.

[163] Homocysteine Studies Collaboration (2002). Homocysteine and risk of ischemic heart disease and stroke: a meta-analysis. JAMA.

[164] Di Angelantonio E, Chowdhury R, Sarwar N, Ray KK, Gobin R, Saleheen D (2009). B-type natriuretic peptides and cardiovascular risk systematic review and meta-analysis of 40 prospective studies. Circulation.

[165] Kistorp C, Raymond I, Pedersen F, Gustafsson F, Faber J, Hildebrandt P (2005). N-terminal pro-brain natriuretic peptide, c-reactive protein, and urinary albumin levels as predictors of mortality and cardiovascular events in older adults. JAMA.

[166] Schneider HJ, Wallaschofski H, Volzke H, Markus MR, Doerr M, Felix SB (2012). Incremental effects of endocrine and metabolic biomarkers and abdominal obesity on cardiovascular mortality prediction. PLoS One.

[167] Kanhai D, Kranendonk M, Uiterwaal C, Graaf Y, Kappelle L, Visseren F (2013). Adiponectin and incident coronary heart disease and stroke. A systematic review and meta‐analysis of prospective studies. Obes Rev.

[168] Smith GD, Ben-Shlomo Y, Beswick A, Yarnell J, Lightman S, Elwood P (2005). Cortisol, testosterone, and coronary heart disease: prospective evidence from the Caerphilly study. Circulation.

[169] Hamer M, Endrighi R, Venuraju SM, Lahiri A, Steptoe A (2012). Cortisol responses to mental stress and the progression of coronary artery calcification in healthy men and women. PLoS One.

[170] Pai JK, Cahill LE, Hu FB, Rexrode KM, Manson JE, Rimm EB (2013). Hemoglobin A1c is associated with increased risk of incident coronary heart disease among apparently healthy, nondiabetic men and women. J Am Heart Assoc.

[171] Gast KB, Tjeerdema N, Stijnen T, Smit JW, Dekkers OM (2012). Insulin resistance and risk of incident cardiovascular events in adults without diabetes: meta-analysis. PLoS One.

